# Quantitative Data for Transcutaneous Electrical Nerve Stimulation and Acupuncture Effectiveness in Treatment of Fibromyalgia Syndrome

**DOI:** 10.1155/2019/9684649

**Published:** 2019-03-04

**Authors:** Merve Yüksel, Şehri Ayaş, Mehmet Tuğrul Cabıoğlu, Derya Yılmaz, Cağrı Cabıoğlu

**Affiliations:** ^1^Alanya Alaaddin Keykubat Education and Research Hospital, Alanya, Antalya, Turkey; ^2^Department of Physical Therapy and Rehabilitation, Faculty of Medicine, Başkent University, Ankara, Turkey; ^3^Department of Physiology, Faculty of Medicine, Başkent University, Ankara, Turkey; ^4^Department of Electrical and Electronics Engineering, Faculty of Engineering, Başkent University, Ankara, Turkey; ^5^Department of Computer Engineering, Engineering Faculty of Başkent University, Ankara, Turkey

## Abstract

*Aim*. To evaluate the effects of acupuncture and transcutaneous electric nerve stimulation (TENS) applications on the quantitative electroencephalography (qEEG) changes and to evaluate their therapeutic effects in patients with fibromyalgia syndrome (FMS). The study included 42 patients with FMS and 21 healthy volunteers. The patients were randomly assigned to two groups (n=21 in each) to undergo either TENS or acupuncture application. In both acupuncture and TENS groups, baseline electroencephalography (EEG) recording was performed for 10 min and, then, TENS or acupuncture was performed for 20 min, followed by another 10 min EEG recording. Baseline qEEG findings of FMS patients in the TENS and acupuncture groups were similar. Delta and theta powers over the frontal region of FMS patients were lower than controls. Theta powers of right posterior region were also lower than controls. In the TENS group, after the treatment, an increase was observed in the alpha power of the left anterior region as well as a decrease in pain scores. In the acupuncture group, an increase was determined in the alpha power of the right and left posterior regions as well as a decrease in pain score after the treatment. The power of low- and moderate-frequency waves on resting EEG was decreased in the patients with FMS. Decreased pain and increased inhibitor activity were found on qEEG after TENS and acupuncture applications. In conclusion, both TENS and acupuncture applications seem to be beneficial in FMS patients.

## 1. Introduction

Fibromyalgia syndrome (FMS) is a complex chronic pain syndrome accompanied by widespread pain with the presence of tender points, fatigue, sleep disorder, and cognitive disorders. Its pathophysiology still remains unclear. Although the theories on the pathophysiology of FMS have initially underlined the pathologies of muscular origin, the origin of pain has recently turned toward processing disorders [1]. It has been demonstrated that chronic painful stimuli in FMS patients lead to neuroplasticity and dysfunction in pain pathways in the central nervous system and that the brain gives different responses to pain [2]. Disseminated hyperalgesia, allodynia, and pain spreading over spinal segments are common in FMS patients. Also, increased sensitivity to visual and auditory stimuli in addition to the presence of pain supports the fact that the underlying problem is pain and/or sensorial. There is a study using functional magnetic resonance imaging in FMS patients which appears to support exaggerated central pain response [3].

Effects of TENS on pain and the gate control mechanism have been known for a long time. Following high-frequency stimulation, increased enkephalins and gamma-aminobutyric acid concentrations, which are endogenous opioids, have been shown, whereas following low-frequency stimulation in the central nervous system, increased beta-endorphin and serotonin concentrations have been demonstrated and the analgesic effect is eliminated, when antagonists are given, suggesting that analgesia is partially provided by these mediators [4, 5].

The history of acupuncture dates back to 3,000 years ago. Currently, it is used as a solution for many health problems. In recent times, its use in diseases with pain symptoms has been gradually increasing. The pain control system is activated as the acupuncture needle is inserted into the point, and an increase is determined in endogenous opioids such as beta-endorphin [6–8], enkephalins [9], and endomorfin [10], as well as in the serotonin [11] concentrations both in the plasma and in the central nervous system.

Quantitative EEG is a neurophysiological method frequently used to evaluate central pain processing [12–14]. In the present study, we aimed to evaluate the effects of acupuncture and TENS applications on the changes in qEEG and to evaluate their therapeutic effects in patients with FMS.

## 2. Materials and Methods

The study protocol was approved by the Başkent University Institutional Review Board and Ethics Committee (no.: KA 11/238). All patients were informed about the study and their consents were obtained prior to their participation in this study. The study was conducted in accordance with the principles of the Declaration of Helsinki.

The study sample consisted of 42 patients in the age group of 21-65 years who were diagnosed with FMS according to the American College of Rheumatology Classification System among those who were admitted to Başkent University, Faculty of Medicine, Department of Physical Therapy and Rehabilitation outpatient clinic, between January 2012 and July 2013. The control group consisted of 21 healthy volunteers. In order to compare the EEG findings of patients with fibromyalgia and healthy individuals, we formed the control group of healthy individuals. Patients with endocrine disorders (i.e., diabetes mellitus and thyroid disease), neurological disorders (i.e., epilepsy and cerebrovascular accident), and chronic renal insufficiency and those receiving antidepressant-antiepileptic drugs were excluded. In addition, patients who were contraindicated for TENS (i.e., pacemaker implantation) were excluded. Age of all participants and time to the onset of symptoms were recorded.

In the patients diagnosed with FMS, severity of pain and fatigue was assessed using Visual Analog Scale (VAS), whereas pain threshold levels of tender points were determined by algometry. Severity of depression was assessed using Beck Depression Inventory (BDI). Severity of disease and quality of life were evaluated by Fibromyalgia Impact Questionnaire (FIQ).

Tender points were detected using digital palpation method, which corresponds to the application of approximately 4 kg pressure at an intensity that would turn the thumb white. Pressure pain threshold (PPT) measurement was performed bilaterally in the tender points using pressure algometry device (JTECH Medical, Midvale, UT, USA). To obtain the total PPT score, PPT values were summed after measurement of PPT in 18 tender points, which are characteristic for FMS.

Current pain and fatigue levels of the patients who were assigned to TENS or acupuncture group were evaluated using VAS prior to EEG. Change in the level of pain was reevaluated using VAS after TENS and acupuncture applications.

Acupuncture was applied on the Houxi (SI 3), Wangu (SI 4), Shenmai (UB 62), Jinggu (UB 64), Shugu (UB 65), and Yintang points for 20 min using a steel needle. We used Kingli sterile acupuncture needles (0.25 mm x 25 mm) in the present study. In addition, TENS was applied using the Endomed CV 405 device (Chattanooga, TN, USA). We placed electrodes of TENS at the T2 and T6 paravertebral level in the upper thoracic region. 70 Hz 100 ms conventional TENS was applied for 20 min. Current intensity of TENS was finely adjusted to create a mild tingling sensation in the patients without causing contraction or discomfort.

A total of 42 patients were randomly assigned to two groups of 21 each to receive either TENS or acupuncture application. Procedures were performed after all participants in these two groups and control group were allowed to rest for 10 min. In the TENS and acupuncture groups, EEG was recorded for 10 min following resting and, then, TENS or acupuncture was performed for 20 min, and EEG was recorded again for 10 min after the application. In the control group, EEG was recorded for 20 min following resting. The EEG recording was performed at Başkent University, Faculty of Medicine, Acupuncture Therapy Unit, Umitkoy, Ankara, using the NicoletOne v32 device (Natus Manufacturing Ltd. Co., Galway, Ireland). The electrodes were placed via a cup electrode in accordance with the international 10/20 electrode system, and each electrode area was filled with electro gel. The electrodes used were Fp1, F3, C3, P3, O1, F7, T3, T5, A1, Fz, Cz, Pz, Fp2, F4, C4, P4, O2, F8, T4, T6, and A2. While placing the cups, the electrodes Fp1 and Fp2 were enabled to be on the correct position. The position of Cz electrode was checked. Electrodes were placed in accordance with the three-point principle. During recording, the device was adjusted at sampling frequency of 500 Hz, band-pass filter interval of 0.5-35 Hz, and amplitude of 100 microvolts. Attention was paid to keep all electrode impedances to be under 5 KOhms. Procedures were performed with closed eyes. The temperature of the room where the EEG recordings and applications were made was 24°C. This room is 35 square meters. Attention was paid to prevent factors such as sound or light which might distract the patients' attention, and each subject was checked to see whether she/he was awake. Five-minute EEGs without artifacts were used for the spectral analysis.

EEG data recorded from each electrode were filtered to obtain frequency components of delta (0.5–4 Hz), theta (4–8 Hz), alpha (8-13 Hz), and beta (13– 30 Hz) EEG bands after removing linear trends. For obtaining powers of EEG bands, the power spectral densities of these filtered signals were calculated by using Fourier transform with 0.25 frequency resolution for each electrode.

### 2.1. Statistical Analysis

Statistical analysis was performed using the SPSS version 16.0 statistical software (SPSS Inc., Chicago, IL, USA). Descriptive data were expressed in mean ± standard deviation (SD) for normally distributed data and median (min-max) for abnormally distributed data. Whether the data were suitable for normal distribution was analyzed by Shapiro-Wilk normality test, while the homogeneity of the variances was evaluated by Levene's test. Delta, theta, alpha, and beta frequencies of each electrode and the four regions were compared using the nonparametric Kruskal-Wallis test and Mann-Whitney* U* test. The Mann-Whitney* U* test was also used for multiple comparisons (*post hoc*) with Bonferroni correction. The electrodes and regions that showed differences in terms of EEG powers and pre- and posttreatment values of the TENS and acupuncture groups were evaluated using the Wilcoxon signed-rank test for abnormally distributed data and using Student's paired test for normally distributed data. The relation between regional alpha, beta, delta, and theta powers before treatment and disease duration, total PPT score, and VAS pain and fatigue scores before treatment were assessed using Spearman's correlation analysis. Clinical data were evaluated by parametric independent two-sample* t*-test for normally distributed data and nonparametric Mann-Whitney* U* test for abnormally distributed data. The age of participants in all groups was evaluated using parametric one-way analysis of variance (ANOVA) and Tamhane's T2 multiple comparison test. The BDI scores were evaluated using chi-square independence test (Yates chi-square). Pre- and posttreatment VAS pain scores were evaluated using nonparametric Wilcoxon* t*-test. A p value < 0.05 was considered statistically significant.

## 3. Results

### 3.1. Demographic and Clinical Findings

In the present study, the mean age of FMS patients in the acupuncture group was 44.6±10.34 years, the mean age of FMS patients in the TENS group was 38.05±11.3 years, and the mean age of the control group was 30.24±6.5 years. The control group was younger than the acupuncture and TENS groups (p<0.05).

The mean FIQ and fatigue scores showed no significant difference between the acupuncture and TENS groups (p>0.05). The mean total PPT score was significantly lower in the TENS group compared to the acupuncture group (80.47±13.36 versus 90.61±12.98, resp.; p<0.05).

The presence of depression in the acupuncture and TENS groups was assessed using the BDI. According to the Turkish validity and reliability study of this scale, the cut-off value was considered as 17 [15]. Accordingly, scores lower than 17 indicate no depression and scores higher than 17 indicate the presence of depression. In our study, depression was found in 12 patients in the acupuncture group and 10 patients in the TENS group, and the results of BDI were similar in both groups (p>0.05).

The median duration of the disease was five (1-20) years in the acupuncture group and four (1-8) years in the TENS group, suggesting no statistically significant difference (p>0.05). The median pain score of FMS patients assessed by VAS before treatment was 5 (1-7) in the acupuncture group and 5 (1-10) in the TENS group, indicating no statistically significant difference (p>0.05).

### 3.2. Quantitative Electroencephalography Data

Electroencephalography data were analyzed by dividing the brain into four quadrants. Accordingly, “Fp1+F3+F7” formed the left anterior region, “Fp2+F4+F8” formed the right anterior region, “P3+T3+T5+O1” formed the left posterior region, and “P4+T4+T6+O2” formed the right posterior region. The sum of the alpha, beta, theta, and delta powers obtained from these regions formed the regional power.

For delta power, evaluation of pretreatment EEGs of the patients in the acupuncture and TENS groups and the bands from four regions in the healthy controls showed a statistically significant difference between the groups for left anterior and right anterior regions (p<0.05, Table 1). A decrease was recorded in delta power of the left anterior and right anterior regions when acupuncture and TENS groups were compared with the control group. Delta power of the left and right posterior regions was found to be weaker in the acupuncture group compared to the control group (p<0.05).

For theta power, pretreatment EEGs of the patients revealed a decrease in theta power of the left anterior, right anterior, and right posterior regions in the acupuncture and TENS groups compared to the healthy control group (p<0.05, Table 2).

For alpha power, when the alpha power obtained from pretreatment EEGs of the patients in the acupuncture and TENS groups was compared with the control group, a decrease was observed in the alpha power of the left and right anterior regions in the acupuncture group (p<0.05, Table 3). Beta power of the right and left anterior regions was found to be significantly lower in the acupuncture group compared to the control group (p<0.05, Table 4).

### 3.3. Comparison between Clinical Findings and Quantitative Electroencephalography Findings

We investigated the correlation of regional alpha, beta, theta, and delta powers obtained from pretreatment baseline EEGs of patient groups with the total PPT score, disease duration, and VAS pain and fatigue scores. In the acupuncture group, a negative correlation was found between pretreatment theta power of the left posterior region and pretreatment pain score (r=-0.456, p=0.038), whereas a positive correlation was found between pretreatment alpha power of the left anterior region and the disease duration (r=0.539, p=0.017). In the TENS group, a positive correlation was found between pretreatment alpha power of the left anterior region and disease duration (r=0.471, p=0.031). However, no significant correlation was found between other regional powers and clinical data.

### 3.4. Effects of Transcutaneous Electric Nerve Stimulation and Acupuncture Applications on Clinical and Quantitative Electroencephalography Parameters

Pain scores using VAS were 5 (1-10) (mean±SD: 5.19±2.20) before TENS application and 3 (0-8) (mean±SD: 2.86±2.01) after TENS application in FMS patients, indicating a statistically significant decrease (p=0.001). The median VAS pain scores in FMS patients were 5 (1-7) (mean±SD: 4.62±1.56) before acupuncture application and 2 (0-9) (mean±SD: 2.60±2.28) after treatment, indicating a decrease in the pain scores after treatment (p=0.003).

When the regional theta power obtained in the qEEG which was recorded after TENS in FMS patients was compared with pretreatment theta power, an increase was observed in the right posterior region (p<0.05, Table 5).

When the regional alpha power obtained in the EEG which was recorded after TENS in FMS patients was compared with its pretreatment alpha power, an increase was observed in the left anterior region and a decrease was observed in the right posterior region (p<0.05, Table 5).

When the regional theta power obtained in the qEEG recorded after acupuncture application was compared with pretreatment theta power in FMS patients, an increase was observed in the right posterior region (p<0.05, Table 5).

When the regional alpha power obtained in the qEEG recorded after acupuncture application was compared with pretreatment alpha power in FMS patients, an increase was observed in the right posterior and left posterior regions (p<0.05, Table 5).

When the regional beta power obtained from EEG recorded after acupuncture application was compared with the pretreatment beta power, an increase was observed in the right posterior and left posterior regions (p<0.05, Table 5).

## 4. Discussion

In the current study, we investigated the association between disease duration, different clinical symptoms of FMS including pain, fatigue, depression, pressure pain threshold, and resting state qEEG brain activity, and effects of TENS and acupuncture treatments. Our study results showed that the main PPT scores were significantly lower in FMS patients in the TENS group compared to the acupuncture group. However, disease duration and other clinical symptoms of FMS were not significantly different in the TENS and acupuncture groups.

In our study, delta and theta powers over the frontal region of FMS patients in TENS and acupuncture groups were lower than controls. Theta powers of right posterior region in both FMS patient groups were also lower than controls. Beta and alpha powers of frontal region in the acupuncture group were lower than the controls. These findings are consistent with previous results obtained by Hargrove et al. [16], indicating that FMS patients had a decrease in the absolute power of low-medium-frequency (delta, theta, and alpha) waves, particularly in the frontal region, whereas there was an increase in the relative power of high-frequency (beta) waves in the frontal/central regions. Another study showed that FMS patients displayed reduced delta activity in right insula and temporal cortices as well as enhanced beta activity in right frontal mid-cingulate and motor areas compared to pain-free controls [17]. Reduced delta activity over the insular cortex and increased beta activity over frontal and mid-cingulate cortices in FMS patients have been interpreted as an increase of excitatory processes in these brain regions during rest due to chronic pain. It also found that these alterations were related to clinical variables in FMS patients: pain duration was significantly associated with reduced delta power over insula, and anxiety and depression score were associated with beta power in frontomedial regions [17]. In contrast, in our study, pretreatment beta power in acupuncture group decreased over the frontal region.

Another study showed that FMS patients exhibited increases in the frontal theta power during resting EEG. Furthermore, theta power was associated with increased tenderness and tiredness score in FMS patients [18]. In our study, neither the disease duration and VAS pain score nor other clinical findings of FMS showed any correlation with qEEG regional powers of FMS patients that exhibited significant differences compared to the controls.

Transcutaneous electrical nerve stimulation is frequently used by health professionals for pain control. The effects of TENS for pain control are explained by the gate control theory, which suggests that electrical stimulation of large diameter afferent fibers inhibits second-order neurons in dorsal horn and prevents impulses carried by small-diameter fibers from being transmitted [19]. Stimulation of opioid receptors has been also proposed as a possible mechanism of action of TENS. It has been shown that high- and low-frequency TENS analgesia was mediated by different opioid receptors, with low-frequency TENS producing analgesia through MU opioid receptor and high-frequency TENS which producing through delta opioid receptors [20]. Both low- and high-frequency TENS have been used for FMS treatment [19, 21, 22]. In our study, TENS-induced alterations in the brain are important findings, since we recorded brain activity. We did not use low-frequency TENS, as alterations in brain activity may be limited by reduced MU opioid receptor binding potential within several regions in brain which are known to play a role in pain modulation [23].

In the present study, we found that TENS treatment resulted in a significant decrease in pain intensity and alpha power over right posterior region of FMS patients, compared to pretreatment level. Alpha waves theoretically reflect the activities of inhibitor neurons [24]. Changes in alpha power have been described as outcome measures after TENS application [25]. Silva et al. [26] confirmed a decrease in slow and fast absolute alpha power during both acupuncture and burst TENS applications compared to the resting state over the parietal region in nerve pain. They reported that decreased alpha power over parietal region can be interpreted as greater attention to painful processes during TENS application. Increased mobilization of attention resources to pain and angry faces evoked greater theta power as well as reduced alpha power in FMS patients than pain-free controls over the parietal region [27]. In our study, reduced alpha power and increased theta power over right posterior region after TENS application can be explained as enhanced attention to one's own pain in FMS patients. However, increased alpha power over left anterior region after TENS application in FMS patients can be related to augmented activities of inhibitor neurons.

In recent years, many studies have been conducted to explain the mechanism of action of acupuncture. Studies on humans and animals have demonstrated that pain control system is activated, as the acupuncture needle is inserted into the point and analgesic, anti-inflammatory, anxiolytic, and sedative efficacies are enabled by increasing endogenous opioids [9, 28, 29] and serotonin [11] concentrations in both the plasma and the central nervous system. It has been increasingly used in recent decades, particularly for the management of pathologies that cause intensive pain and, therefore, it is recommended also for FMS. Despite wide acceptance among patients and health professionals, evidences on the efficacy of acupuncture in the treatment of FMS are yet lacking. Vas et al. [30] randomized 156 FMS patients to either real or sham acupuncture groups and concluded that it might provide evidence on the efficacy of acupuncture as a therapeutic option for FMS in patients with and without severe depression. Similar to our study design, in another study, Martin et al. [31] randomized 50 fibromyalgia patients to either real or sham acupuncture groups and found that acupuncture significantly resolved fibromyalgia symptoms, compared to placebo, and that the most notable resolution was observed in fatigue and anxiety during follow-up.

In the present study, we used Houxi (SI 3), Wangu (SI 4), Shenmai (UB 62), Jinggu (UB 64), Shugu (UB 65), and Yintang points. It is known that Houxi (SI 3) and Shenmai (UB 62) are the eight confluent points and they are affective in diseases with pain symptoms in the neck, shoulder, thoracic, and lumbar regions. The Wangu (SI 4) point is used for painful conditions in the Yuan-Primary point of SI meridian and shoulder-neck region. The Jinggu (UB 64) point is used for painful conditions in the Yuan-Primary point of UB meridian and the neck-lumbar region. The Shugu (UB 65) point is used for painful conditions in the neck-lumbar region and lower extremities [32]. The Yintang point is used for psychological alterations such as anxiety and depression [33–35]. Since the majority of painful points with pressure are found in the neck, back, lumbar region, and lower extremities in FMS patients, acupuncture points that are specifically effective on these points were chosen. Due to the presence of psychological alterations in FMS patients [35–37], Yintang point, which is effective in this condition, was chosen.

In the present study, in the acupuncture group, increased alpha power of the right and left posterior regions, increased theta power of the right posterior region, and increased beta power of the right and left posterior regions were observed after treatment. According to the pain scores before and after acupuncture in this group, a decrease was observed in pain score, but an increase was found in the alpha-wave power. Increased alpha power may be related to the suppression of pain sensation by activation of inhibitor neurons in the central nervous system due to the stimulation of pain control system, which was excited by acupuncture application [24]. The literature review, however, revealed no publication on the change in EEG caused by acupuncture in FMS patients. Nevertheless, increased alpha power on EEG record was found as the result of acupuncture applied to the pericardium 6 point in healthy individuals [38].

Increased delta and theta activities in acute pain were attributed to the negative emotional response to cold compress, whereas increased beta activity was attributed to hypersensitivity to tonic pain [39]. In addition, increased delta and beta powers may be often related to emotional response to pain [40]. In our study, increased beta power following acupuncture might have resulted from negative emotional responses of the patient to the first-time acupuncture procedure. It was also reported that there might be an increase in beta 2 activity due to muscle contraction [14]. Hence, the muscle activity, which was stimulated by acupuncture-related pain, might have increased the beta activity.

There are some limitations to this study. Small sample size and young mean age of the control group can be regarded as the main limitations. In addition, we were unable to assess for how long altered EEG activity following TENS and acupuncture applications was maintained. Therefore, further large-scale, randomized, prospective studies are required to confirm these findings.

## 5. Conclusions

In conclusion, our study results showed a decrease in the power of low-medium-frequency waves in the EEGs of FMS patients with high pain scores. Decreased pain scores and increased alpha power over anterior region after TENS and acupuncture application in FMS patients can be associated with augmented activities of inhibitor neurons. Considering pain scores and EEG findings, we suggest that both TENS and acupuncture applications seem to be beneficial in FMS patients.

## Figures and Tables

**Table 1:** Comparison of regional delta power obtained from pretreatment electroencephalography recordings of the patients in the acupuncture and transcutaneous electric nerve stimulation groups with the control group.

Region	Acupuncture [1]	TENS [2]	Control [3]	Kruskal-Wallis Test		Mann-Whitney U Test (post-hoc)			
				Chi square	p	U and Z values			p
						1 vs. 3	2 vs. 3	1 vs. 3	2 vs. 3
Left anterior	33.75	38.00	63.49	14.115	0.001	U=54	U=86	≤0.001	0.006
	(8.67-94.88)	(1.18-156.98)	(32.66-132.91)			Z=-3.684	Z=-2.748		

Right anterior	30.96	31.095	66.765	10.027	0.007	U=80	U=92	0.003	0.009
	(9.06-118.51)	(11.43-162.62)	(6.83-108.66)			Z=-2.924	Z=-2.573		

Left posterior	84.905	88.945	169.785	6.406	0.041	U=93	-	0.011	-
	(48.36-266.14)	(1.45-297.1)	(27.3-258.68)			Z=-2.543			

Right posterior	82.81	96.535	169.4	12.206	0.002	U=61	-	0.001	-
	(3.86-282.13)	(40.03-275.62)	(79.79-291.95)			Z=-3.479			

TENS, transcutaneous electric nerve stimulation.

Data are presented as median (minimum–maximum).

**Table 2:** Comparison of regional theta power obtained from pretreatment electroencephalography recordings of the patients in the acupuncture and transcutaneous electric nerve stimulation groups with the control group.

Region	Acupuncture [1]	TENS [2]	Control [3]	Kruskal-Wallis Test		Mann-Whitney U Test (post-hoc) Test			
				Chi square	p	U and Z values			p
						1 vs. 3	2 vs. 3	1 vs. 3	2 vs. 3
Left anterior	4.61	7.42	12.38	21.296	≤0.001	U=39	U=96	≤0.001	0.002
	(2.48-67.56)	(0.26-54.51)	(7.02-31.83)			Z=-4.566	Z=-3.132		

Right anterior	5.66	6.55	12.11	18.126	≤0.001	U=66	U=85	≤0.001	0.001
	(2.64-96.14)	(1.54-18.73)	(1.73-42.71)			Z=-3.887	Z=-3.409		

Left posterior	19.81	25.53	36.24	7.228	0.027	-	-	-	-
	(13.26-268.65)	(0.37-147.12)	(5.78-104.04)						

Right posterior	19.38	21.38	37.77	12.929	0.002	U=94	U=103	0.001	0.003
	(0.76-210.78)	(9.94-102.13)	(17-96.67)			Z=-3.182	Z=-2.956		

TENS, transcutaneous electric nerve stimulation.

Data are presented as median (minimum–maximum).

**Table 3:** Comparison of regional alpha power obtained from pretreatment electroencephalography recordings of the patients in the acupuncture and transcutaneous electric nerve stimulation groups with the control group.

Region	Acupuncture [1]	TENS [2]	Control [3]	Kruskal-Wallis Test		Mann-Whitney U Test	
						(post-hoc)	
				Chi square	p	U and Z values for 1 vs. 3	p
Left anterior	3.54	4.84	8.35	18.295	≤0.001	U=52	≤0.001
	(1.18-14.86)	(0.17-21.9)	(2.96-18.47)			Z=-4.239	

Right anterior	3.83	4.38	9.21	9.873	0.007	U=101	0.003
	(1.98-20.47)	(1.35-13.83)	(0.71-23.76)			Z=-3.006	

Left posterior	27.745	32.75	60.56	3.226	0.199	-	-
	(10.83-140.8)	(11.86-141.99)	(5.69-249.9)				

Right posterior	43.8	46.39	77.77	4.422	0.110	-	-
	(0.53-216.26)	(2.06-129.23)	(13.76-214.82)				

TENS, transcutaneous electric nerve stimulation.

Data are presented as median (minimum–maximum).

**Table 4:** Comparison of regional beta power obtained from pretreatment electroencephalography recordings of the patients in the acupuncture and transcutaneous electric nerve stimulation groups with the control group.

Region	Acupuncture [1]	TENS [2]	Control [3]	Kruskal-Wallis Test		Mann-Whitney U Test	
						(post-hoc)	
				Chi square	p	U and Z values for 1 vs. 3	p
Left anterior	4.42	6.23	11.93	10.292	0.006	U=94	0.001
	(1.83-15.62)	(0.28-50.6)	(4.68-96.73)			Z=-3.182	

Right anterior	5.45	6.02	11.35	8.083	0.018	U=113	0.007
	(2-28.25)	(2.33-28.01)	(1.16-77.66)			Z=-2.704	

Left posterior	25.12	25.85	33.87	4.53	0.104	-	-
	(13.4-71.51)	(11.2-109.55)	(6.53-125.84)				

Right posterior	28.44	26.47	31.32	4.005	0.135	-	-
	(0.82-57.87)	(9.98-96.30)	(17.43-168.54)				

TENS, transcutaneous electric nerve stimulation.

Data are presented as median (minimum–maximum).

**Table 5:** Comparison of regional powers before and after treatment in the acupuncture and transcutaneous electric nerve stimulation groups.

Region			Before treatment	After treatment	Wilcoxon Signed-Rank Test	
			Median (Min–Max)	Median (Min–Max)	Z	p
Delta	Acupuncture	Left anterior	33.75 (8.67-94.88)	36.88 (9.65-119.19)	-	-
		Right anterior	30.96 (9.06-118.51)	38.26 (15.23-127.02)	-	-
		Left posterior	84.905 (48.36-266.14)	92.12 (44.13-346.49)	-	-
		Right posterior	82.81 (3.86-282.13)	83.605 (37.56-320.62)	-	-
	TENS	Left anterior	38.00 (1.18-156.98)	47.04 (14.07-126.07)	-	-
		Right anterior	31.095 (11.43-162.62)	26.21 (12.54-97.5)	-	-
		Left posterior	88.945 (1.45-297.1)	104.4 (62.3-270.2)	-	-
		Right posterior	96.535 (40.03-275.62)	83.66 (55.86-249.22)	-	-

Theta	Acupuncture	Left anterior	4.61 (2.48-67.56)	5.05 (2.18-34.75)	-	-
		Right anterior	5.66 (2.64-96.14)	5.25 (2.21-57.31)	-	-
		Left posterior	19.81 (13.26-268.65)	27.28 (8.56-166.79)	-	-
		Right posterior	19.38 (0.76-210.78)	26.28 (2.29-132.44)	-2.033	0.042
	TENS	Left anterior	7.42 (0.26-54.51)	6.33 (2.1-60.99)	-	-
		Right anterior	6.55 (1.54-18.73)	5.47 (3.21-20.25)	-	-
		Left posterior	25.53 (0.37-147.12)	26.2 (11.34-68.46)	-	-
		Right posterior	21.38 (9.94-102.13)	23.79 (10.97-60.34)	-2.213	0.027

Alpha	Acupuncture	Left anterior	3.54 (1.18-14.86)	3.525 (1.18-8.48)	-	-
		Right anterior	3.83 (1.98-20.47)	4.955 (2.11-8.74)	-	-
		Left posterior	27.745 (10.83-140.8)	61.25 (10.69-160.38)	-2.725	0.006
		Right posterior	43.8 (0.53-216.26)	59.11 (1.78-240.48)	-3.285	0.001
	TENS	Left anterior	4.84 (0.17-21.9)	4.96 (2-25.89)	-2.069	0.039
		Right anterior	4.38 (1.35-13.83)	4.46(2.17-9.96)	-	-
		Left posterior	32.75 (11.86-141.99)	47.04 (10.34-200.33)	-	-
		Right posterior	46.39 (2.06-129.23)	40.34 (6.99-170.15)	-2.696	0.007

Beta	Acupuncture	Left anterior	4.145 (1.83-15.62)	6.735 (1.71-21.46)	-	-
		Right anterior	5.335 (2-28.25)	8.07 (2.57-15.66)	-	-
		Left posterior	24.57 (13.4-71.51)	29.22 (19.35-66.14)	-2.389	0.017
		Right posterior	28.195 (0.82-57.87)	31.97 (2.73-73.99)	-2.837	0.005
	TENS	Left anterior	6.23 (0.28-50.6)	6.33 (2.1-60.99)	-	-
		Right anterior	6.02 (2.33-28.01)	5.47 (3.21-20.25)	-	-
		Left posterior	25.85 (11.2-109.55)	27.03 (11.34-120.29)	-	-
		Right posterior	26.47 (9.98-96.30)	23.79 (10.97-60.34)	-	-

## Data Availability

The derived power data from raw EEG of each electrode for all patients used to support the findings of this study are available from the corresponding author upon request.
